# The Physiology of Teaching: Getting the Balance right

**DOI:** 10.15694/mep.2020.000196.1

**Published:** 2020-09-15

**Authors:** Gabrielle Brand

**Affiliations:** 1 Monash University

**Keywords:** living curriculum, student engagement, curiosity, reflection, social learning, collaborative learning, communication, interprofessional

## Abstract

This article was migrated. The article was marked as recommended.

Using three human body systems as a metaphor, this paper will discuss how the art of teaching, including the conditions in which students thrive can remedy longstanding curriculum diseases. Based on the concept of a living curriculum, the importance of curiosity, exposing vulnerability, social and collaborative learning, communication and reflective learning processes in transforming teaching and inspiring learning will be explored.

## Introduction

A living curriculum is dynamic, reciprocal, experiential, authentic, reflective, creative and sustainable. Whilst exploring this concept it reminded me of living body systems and how when working together in homeostatic harmony there is a magic that happens - just like when deep and transformative learning occurs in our students and ourselves. So, if the curriculum is alive then it is also susceptible to abnormalities (deviations to normal that we often refer to as disease). This reminded me of Abrahamson (1978) seminal work which he first published in 1978 in the Journal of Medical Education where he describes common diseases of the curriculum. I must admit I was a little disheartened that after 40 years these diseases still exist - and in some cases it may be terminal! So how do we cure these curriculum diseases or at the very least relieve some of the symptoms?

In this paper, I will draw from three human body systems (cardiovascular, digestive & nervous) and use these as a metaphor to illustrate some of the most common curriculum diseases. In addition, I will share some novel innovations as potential remedies to enhance the scholarship of learning and teaching.

## The Cardiovascular System

The first system is the cardiovascular system that centers around the heart, the engine room of the body. The heart relates to your passion and enthusiasm for teaching which has the potential to spark curiosity in previously dark or undiscovered parts of our students’ minds. Cardiovascular diseases are the most common cause of death globally accounting for 30% worldwide. So it is not surprising that *Curriclosclerosis* is a common disease that sees hardening of the curriculum arteries. For example, a narrowing stenosis can be triggered by outdated teaching practices that are based in conformity, one size fits all approach to produce passive, docile learners. In the worst-case scenario, left without treatment, *curriclosclerosis* can permeate the whole faculty and cause a hardening of the whole heart of a once inspired teacher. This may cause a cardiac arrest which leads to a loss of output and is the leading cause of a soulless curriculum. A weakening of the heart can cause *Curriculum Malaise* (defined as a general feeling of discomfort, illness, or unease whose exact cause is difficult to identify). I often experience this myself and see this ailment in my academic colleagues at the end of the year. So, what can we do to remedy this?

The best teachers are contagious, they love learning and role model curiosity to students. If teachers are interested, connect and find meaning in what they are teaching, they will know how to spark curiosity in their students. Recently, I have been trying what my colleagues Bearman and Molloy (2017) have termed intellectual streaking: the value of teachers exposing minds (and hearts). They argue that we ask our students to be vulnerable all the time, to critique or perform a skill for the first time in front of their peers while we assess them. However, we don’t reciprocate this vulnerability. They suggest that teachers should take more risks, expose uncertainties, internal dilemmas, human emotions, thought processes, times they have tried and failed. This is scary and confronting as it relocates power and status of traditional teaching boundaries.

The first time I tried intellectual streaking was during an online forum where I presented some short literacy pieces as a stimulus for students to explore and reflect on death and dying. I asked: How do you feel when reading this literature piece? Did any part of the writing touch or connect with your life professionally or personally?

I decided to set the tone and start off the online forum by posting my own personal response to an excerpt from Marion Coutts (2016) book’The Iceberg’ where she recounts going out to a party after her husband’s death:

At a party someone takes my arm and whispers to me, ‘Strong Woman.’ Dear God. My magic vanishes. My power dissolves like powder in water. Weakness is in those nattering companionably all around me. I want please to be one of the weak. The weak are held close and given tea. They are hugged and warmed by the fire. The strong are revered but kept at a distance. They live outside the village.

I put up and took down my personal response three times. Exposed and vulnerable, I wrote:

This piece reminded me of when my first patient died. It was during my fist ever night shift at a major hospital, I was 21 years old. My response to this event was rational and controlled (just as I was trained), my professional mask tightly secured. As I sat debriefing with the stern military trained night manager who commended my control, I realised that my resistance to show the overwhelming emotion I felt inside was directly related to my own story; a story that valued objectivity and rational thought over subjectivity and human emotion. I went home and cried. I am both fierce and fragile and in order to honour my humanness, I must learn how to stand in the tension of these two opposing sides of myself.

The risk paid off, I can’t describe the difference in quality of the posts by students compared to previous years, it was remarkable. The online forums were so reflectively rich and thoughtful.

## The Digestive System

The process of digestion has many stages, it starts at the mouth to breakdown food into smaller and smaller components until the nutrients can be absorbed and the waste product are eliminated. What are the essential nutrients your students’ needs to learn? Are they going to absorb and metabolize this knowledge or eliminate it (and in a predominantly outcomes based curriculum this usually occurs directly after assessment and exams). The digestive system reminds us that learning is a process, not an outcome. Scaffolding through sequencing learning experiences so the overcrowded curriculum content can be digested into manageable smaller components is essential for potent learning and long-term knowledge translation. If this does not occur a case of information overload with too much content can cause *Curriculum constipation* *.*

What would you prescribe? A predominately outcomes based curricula negates living curricula concepts. As Rees (2004) asserts there is problem with control when the effectiveness of educational experiences is determined by the degree of match between the product of the learning experience and the pre-determined learning outcomes. If teachers on the ground are not connected with the content they are teaching, their skill set is diminished and they may feel disempowered to do their job. In contrast, a process driven curriculum focuses on interaction and relationship between the teacher and student and allows for organic, responsive learning through collaborative decision making and meaning making that places the learning in the context of what the student needs at that particular time.

This is not a new concept, Aristotle described it as an intellectual virtue of ‘phronesis’ or more recently referred to as ‘practical wisdom’ ( Schwartz and Sharpe, 2006) that encompasses ‘thinking in action’. It encourages students to draw and connect meaning from their own experience and does not limit educators to teach in a recipe like formulas that privilege content acquisition and the security of certainty. A learning and teaching approach that values openness to unexpectedness allows teachers to observe and respond more naturally and creates a safe learning environment for students to be brave enough to ask why?

Inspired by Lara Varpio’s (2018) concept of creating ‘intellectually nimble’ health professionals that are comfortable with feeling uncomfortable. I have designed a series of Depth of Field reflective learning resources that use visual methodologies like photo-elicitation techniques ( Brand *et al.*, 2016), MRI art ( Brand *et al.*, 2019), and more recently exploring visual narratives co-designed with health care consumers to create conditions for students to practise reflective learning processes. For example, by providing no prior context and deliberately juxtaposing photographs with contrasting narrative based scenarios, I have found student experience an uncomfortable cognitive discrepancy that is key to creating the tension required for transformational learning to occur ( Mezirow, 2000). This lies at the heart of facilitating health professions students’ tolerance of uncertainty/ambiguity, which refers to the way we perceive and process information when confronted by an array of unfamiliar, complex, or incongruent clues ( Budner, 1962; Furnham and Ribchester, 1995), a critical future-protective workplace skill in an evolving health care system.

## The Nervous System

The Brain collects and processes information. The left side of the brain is logical while the right side is creative. Communication between the 2 sides of the brain are essential in reflective practice and particularly important in teaching health professions students to link theory (classroom) with practice (clinical setting). The nervous system relates to the importance of communication and feedback from students, each other and faculty. If the nervous system does not work, the communication channels are cut off resulting in *Curriculoplegia.* This lack of communication can be local or systematic and affect the curriculum horizontally (two units taught simultaneously) or vertically (units taught over years). Communication is reciprocal (just like the living curriculum), a 2 way process of active knowledge construction which can be achieved through social connections and collaborative learning. I draw from Goodson and Gill’s (2011) work where they suggest that this occurs through the dialogic encounter (conversations we have with others) which invoke a ‘third voice’ which have the potential to transform understandings, create new perspectives or ways of seeing the world which changes a person’s course of action. At its essence it is the basis of lifelong learning.

One of my favourite Educational Philosophers was John Dewey (1957) who stated:

“We naturally remember what interests us and because it interests us. The past is recalled not because of itself but because of what it adds to the present. Thus the primary life of memory is emotional rather than intellectual and practical... To revive it and revel in it is to enhance the present moment with new meanings, a meaning different from that which actually belongs either to it or to the past... the conscious and truly human experience... comes when it is talked over and re-enacted.... into a whole meaning.”

Fostering meaningful collaborative and social learning between students is a potent teaching strategy. In Australia, the majority of our health profession courses have changed to graduate entry requirements whereby we have more mature students with life experiences and rich worldview and perspectives. So why don’t we utilize this untapped resource?

As an educator, I wanted to create a space for this ‘third voice’ to emerge to teach students about the importance of interprofessional team work and practice (including some of the barriers and challenges). I teach an array of post graduate health professionals so I decided to draw from their previous life and career experiences and use the concept of the human library. The Human Library is a global movement for social change. It is a place where real people are on loan to readers (instead of a book). It is designed to build positive framework for conversations that can challenge stereotypes and prejudices through dialogue and offer a place where difficult questions are expected, appreciated and answered.

So I partnered an emergency physician with a chiropractor, a nurse with a doctor, a biomedical scientist with a psychologist and a social worker with an intern. I asked them to preflect (before the conversation) and write down their initial and honest reactions to their partners professions. Then they met for a conversation and submitted a post conversation reflection to explore and describe how their views had changed. To my surprise, the emergency physician did not think the chiropractic was a ‘quack’ anymore and learned that there was sound scientific base to their training and the nurse learned that the doctor really does value her clinical reasoning skills and welcomes their advice. This novel innovation debunked some of the unexplored assumptions and led to new understandings of their health professional colleagues.

## Conclusion

In conclusion, we need to collectively make sure we prevent one of the worst disease diagnoses *curriculum ossification* which is defined as a tendency toward or state of being moulded into a rigid, conventional, sterile, or unimaginative condition - the antithesis of how the human body and living curriculum work. So, be like the living body that works together as a whole to create a curriculum that fosters learning environments that Sir Ken Robinson (2006) says “set the climate control of possibility”.

## Take Home Messages

• A living curriculum is experiential, authentic, reflective, creative and dynamic and can remedy long standing curriculum diseases.
• Novel innovation in health care education including intellectual streaking, visual methodologies designed to enhance tolerance of ambiguity and fostering collaborative and social learning in health professions students has the potential to transforms teaching and inspire learning.

## Notes On Contributors

Gabrielle Brand PhD is an Associate Professor at Monash University. She is a passionate teacher and qualitative researcher with a special interest in narrative medicine, interprofessional and reflective learning. ORCiD: https://orcid.org/0000-0001-6606-6721
