# Supplementary material for: The Physiology of Teaching: Getting the Balance right
Source: MedEdPublish (2016). 2020 Sep 15;9:196. [Version 1] doi: 10.15694/mep.2020.000196.1 (PMC10699381; doi:10.15694/mep.2020.000196.1)
Supplement: Peer review history

# Reviewer report: Reviewer response for version 1

**DOI:** 10.21956/mep.20153.r31134

**Published:** 2020-12-08

**Authors:** Ken Masters

**Affiliations:** 1 Sultan Qaboos University

This review has been migrated. The reviewer awarded 4 stars out of 5 An interesting read in which three physiological systems are used as metaphors to discuss problems that may occur in the medical curriculum.Good food for thought. And I think I have learned my new favourite term: “Curriculum constipation”, although I shall need to be careful when and how I use it :-)Although the analogy does work (as long as one does not stretch it too far), there is a missing element: the whole patient, the whole set of influences on the student. Breaking down the problem into the “systems” runs the risk of isolating these components, and we don’t see the whole complex patient. We know that it is only when we see the whole patient that we can train students with complex cases that have no clear-cut answer. When we work with systems, then there is a risk that these systems become silos, and we know that a great difficulty is to get students to integrate across the silos; our view of the student’s education also needs to integrate across all of these. Just as we would like to see students grappling with patient cases, so we need to step back and look at the student’s education as a whole. So, how does the medical school ensure that the entire body: the faculty, the administration, the technical support and the student all integrate and function as a coherent whole with a common purpose? Although this is hinted at in some places in the paper, it does need further exploration and explicit addressing.I think that the analogy would be strengthened if some insight into that could be given. Nevertheless, a fruitful read.

Reviewer Expertise:

NA

No decision status is available

# Reviewer report: Reviewer response for version 1

**DOI:** 10.21956/mep.20153.r31133

**Published:** 2020-09-20

**Authors:** Subha Ramani

**Affiliations:** 1 Harvard Medical School, Brigham and Women's Hospital

This review has been migrated. The reviewer awarded 5 stars out of 5 I completely enjoyed reading this article which likens the art of teaching to physiology using 3 organ systems. It is very creative to think of a curriculum as a living organism and problems with teaching and learning as diseases. The spectrum between physiology and pathology is quite apt for problems related to curricula and their impact. As the author states, if curricular challenges are viewed as diseases, educators could then apply educational principles, formulate a different diagnosis of the problem and then develop solutions. Very clever approach that describes pathology such as curriculosclerosis, curriculum malaise as moderate and severe problems of the heart of curricula. The heart does mirror the emotions involved in teaching and learning such as passion, enthusiasm etc. Other educators have talked about acknowledgement of teacher limitations as establishing a safe learning climate. Here the author similarly talks about intellectual streaking as a strategy to promote curiosity. Dr. Brand applies digestive principles to the process of deep learning and emphasizes the alignment between the teaching process and outcomes, ability to deal with uncertainty and openness to unexpectedness. Finally, the author uses nervous system analogies to discuss bidirectional communication and feedback between faculty and students.This article views teaching and learning through an innovative organ-system based approach. All health professions educators would enjoy reading this paper and able to apply many of these principles to reflect on their own educational practice.

Reviewer Expertise:

NA

No decision status is available
